# Diammonium Glycyrrhizinate Ameliorates Obesity Through Modulation of Gut Microbiota-Conjugated BAs-FXR Signaling

**DOI:** 10.3389/fphar.2021.796590

**Published:** 2021-12-21

**Authors:** Yun Li, Huiqin Hou, Xianglu Wang, Xin Dai, Wanru Zhang, Qiang Tang, Yue Dong, Chen Yan, Bangmao Wang, Zhengxiang Li, Hailong Cao

**Affiliations:** ^1^ Department of Gastroenterology and Hepatology, General Hospital, Tianjin Medical University, Tianjin, China; ^2^ Department of Pharmacy, General Hospital, Tianjin Medical University, Tianjin, China

**Keywords:** diammonium glycyrrhizinate, obesity, gut microbiota, bile acids, farnesoid X-activated receptor

## Abstract

Obesity is a worldwide epidemic metabolic disease. Gut microbiota dysbiosis and bile acids (BAs) metabolism disorder are closely related to obesity. Farnesoid X-activated receptor (FXR), served as a link between gut microbiota and BAs, is involved in maintaining metabolic homeostasis and regulating glucose and lipid metabolism. We previously reported that diammonium glycyrrhizinate (DG) could alter gut microbiota and prevent non-alcoholic fatty liver disease. However, it remains ambiguous how DG affects the gut microbiota to regulate host metabolism. In this present study, 16S rRNA Illumina NovaSeq and metabolomic analysis revealed that DG treatment suppressed microbes associated with bile-salt hydrolase (BSH) activity, which, in turn, increased the levels of taurine-conjugated BAs accompanied by inhibition of ileal FXR-FGF15 signaling. As a result, several obesity-related metabolism were improved, like lower serum glucose and insulin levels, increased insulin sensitivity, few hepatic steatosis and resistance to weight gain. Additionally, decreased level of serum lipopolysaccharide was observed, which contributed to a strengthened intestinal barrier. The effect of DG on weight loss was slightly enhanced in the antibiotics-treated obese mice. Collectively, the efficacy of DG in the treatment of obesity might depend on gut microbiota-conjugated BAs-FXR axis. Hence, it will provide a potential novel approach for the treatment of obesity.

## Introduction

Obesity, mainly caused by genetic and environmental factors, is a worldwide epidemic metabolic disease characterized by excessive fat accumulation. It not only affects body appearance but also contributes to many chronic inflammatory diseases, such as type 2 diabetes, hepatic steatosis and cardiovascular diseases (Petrick et al., 2020; Scheithauer et al., 2020; Agüera et al., 2021). However, the global prevalence of obesity is immensely high and alarming projections suggest that more than one billion people will be obese by 2030 (Kelly et al., 2008; Finucane et al., 2011). Therefore, it is of great importance to explore the mechanism of obesity for search novel treatments and preventive strategies.

It is gradually acknowledged that gut microbiota make a great effect on obesity mainly by regulating energy, acquiring nutrient and storing fat (Scarpellini et al., 2010; Zhang et al., 2010). With the progression of obesity, the composition of gut microbiota can make a change. Generally, the obesity performs an increased ratio of the major phyla Firmicutes/Bacteroidetes in gut microbial composition and injury of intestinal barrier (Ley et al., 2005; Winer et al., 2016; Bona et al., 2021). Evidence suggested that germ-free (GF) mice had significantly lower weight and were protected from fat accumulation than that of conventional mice despite a higher caloric intake (Turnbaugh et al., 2008). In addition, the obese phenotypes were transferred after the gut microbiota from obese donors were transplanted into GF recipients (Bäckhed et al., 2007). Consistently, adiposity and glucose metabolism have been improved in the antibiotics-treated obese mice (Shin et al., 2014).

Bile acids (BAs), a class of cholanic acid derivatives as well as the end products of cholesterol catabolism, are synthesized by the liver (Schneider et al., 2018). BAs, as pleiotropic signaling molecules, regulate its’ own homeostasis, affecting the metabolism of glucose, lipids and energy, and thus participate in the formation of obesity by binding to nuclear receptors such as the farnesoid X-activated receptor (FXR) and takeda G-protein-coupled receptor 5 (TGR5) (Cipriani et al., 2010; Chiang, 2013; Yuan and Bambha, 2015). While BA-mediated stimulation of FXR, mainly distributed in the liver and small intestine (most in ileum), is the major pathway of BA action (Schneider et al., 2018). Negative feedback inhibition of FXR on BA enterohepatic circulation is one of the main mechanisms to maintain BA homeostasis (Schneider et al., 2018). When FXR is activated in ileum, it immediately induces the expression of fibroblast growth factor 15 (FGF15), which, a hormone, subsequently enters the liver via portal vein bloodstream, where it binds with fibroblast growth factor receptor 4 (FGFR4) and inhibits the expression of BA-synthetic rate-limiting enzyme cholesterol 7α hydroxylase (CYP7A1) (Chiang, 2013; Kliewer and Mangelsdorf, 2015). Studies in tissue-specific FXR deficient mice showed that ileum FXR activation inhibited CYP7A1-mediated BA synthesis, which is more powerful than hepatic FXR activation (Kim et al., 2007; Kong et al., 2012). Emerging evidence denoted that inhibition of intestinal FXR-FGF15 signaling resulted in prevention against high fat diet (HFD)-induced obesity and liver steatosis (Jiang et al., 2015a; Degirolamo et al., 2015). Meanwhile, the degree of intestinal FXR activation or inhibition varies in distinct BAs (Lu et al., 2000; Ding et al., 2015; Schneider et al., 2018).

Gut microbiota owns ability to biotransform BAs into a variety of forms, which affect the activation state of FXR as well as expression of its’ downstream molecules, and thus modify BA signaling function to regulate host metabolism (Joyce et al., 2014; Ridlon et al., 2014; Schneider et al., 2018). Primary BAs, generated from cholesterol in liver, are drained into intestinal tract by conjugating with either glycine or taurine, later, these conjugated BAs suffers to deconjugation using bile salt hydrolase (BSH), followed by a serious of reaction including dehydroxylation, differential isomerization and oxidation to produce secondary BAs under metabolic action of gut microbiota in distal small intestine and colon (Staley et al., 2017; Guzior and Quinn, 2021). Study found that intestinal GF mice can resist obesity brought by HFD feeding through FXR-dependent approaches with attenuation of BSH activity (Li et al., 2013; Sayin et al., 2013). Further, antibiotic treatment leads to significant changes in gut microbiota inducing huge alteration of tauro β-muricholate (T-βMCA), a naturally FXR antagonist, and prevents obese mice against adiposity (Sayin et al., 2013; Jiang et al., 2015a).

Diammonium glycyrrhizinate (DG), a binary ammonium salt of glycyrrhizic acid, which is the main saponin and effective component extracted from the Traditional Chinese Medicine of licorice root, possesses various activities such as anti-inflammatory, antiviral and immune regulation (Ochi et al., 2016; Pang et al., 2016). Furthermore, modern pharmacological studies also have shown that licorice root could resist fatty liver and obesity (Madak-Erdogan et al., 2016; Liou et al., 2019). Except to the traditional functions of reducing enzyme, anti-inflammation and improving liver steatosis, some recent studies have associated the anti-obesity effect of DG treatment in mice model with the changes of gut microbiota, which also have been observed in our previous study (Li et al., 2018; Yu et al., 2018; Qiu et al., 2019). However, the underlying specific mechanisms tied with the metabolic benefits brought by DG through regulating gut microbiota remains unclear.

This current study discovered that DG treatment first increased the levels of taurine-conjugated BAs by suppressing the abundance of BSH related microbes in intestine of mice with obesity, which further resulted in restraint of ileal FXR-FGF15 axis to bring several obesity-related metabolic improvements like lower serum glucose and insulin levels, increased insulin sensitivity, few hepatic steatosis and resistance to weight gain. Second, dropped the serum lipopolysaccharide (LPS) levels and enhanced intestinal barrier by declining LPS-producing genera. The results of this study point that the improvement of obesity by DG supplement is mechanically related to changes in gut microbiota, FXR signal transduction and BA synthesis.

## Materials and Methods

### Animals and drug Treatments

Four-week-old C57BL/6J male mice (*n* = 40) weighting 20 ± 2 g were purchased from Beijing HuaFuKang Bioscience Co., Ltd. (Beijing, China). All mice were maintained under specific-pathogen-free (SPF) conditions at Tianjin Medical University animal center with a controlled environment (temperature, 22 ± 2°C; humidity, 45 ± 5%; 12 h/12 h light/dark cycle). All experimental procedures were approved by the local Animal Care and Use Committee of Tianjin Medical University.

Prior to the beginning of respective treatment, all animals were fed a normal chow diet and received sterilized water at libitum for 1 week during acclimatization. Thereafter, 40 mice were randomly distributed into 5 groups of 8 mice each and every group were placed in two cages: control group (NCD) that obtained normal chow diet; normal chow diet with DG group (NCDG) that obtained chow diet with 150 mg/kg of DG intraperitoneally injected every other day; a high fat diet group (HFD) and high fat diet with 150 mg/kg DG intraperitoneal injection group (HFDG); The last group (HFDGA) received high fat diet with 150 mg/kg DG intraperitoneal injection under antibiotic cocktail drinking water instead of sterile water. In the corresponding control groups, an intraperitoneal injection of same volume of vehicle (sterile saline, 0.3 ml) were performed every other day on NCD and HFD groups.

DG was dissolved in sterile saline, its dosage and administration (150 mg/kg, intraperitoneal injection on alternate days) were chosen referenced on our previous studies (Li et al., 2018). The normal chow diet was formulated with 10 E% fat, 20 E% protein, and 70 E% carbohydrates, 3.85 kcal/g contained (H10010, Research Diets, Peking, China). While high fat diet was formulated with 20 E% carbohydrate, 20 E% protein, and 60 E% fat, totally 5.24 kcal/g contained (H10060, Research Diets, Peking, China). The antibiotic cocktail drinking water was renewed every day with a mixture of 200 mg/L ampicillin, metronidazole, neomycin and 100 mg/L of vancomycin (Li et al., 2019). DG was kindly supplied by Chia Tai Tianqing pharmaceutical Company (Jiangsu, China). Antibiotics were purchased from Sigma Aldrich (United States). Weekly body weight was monitored throughout the experiment lasting 14 weeks.

### Tissue Collection

At the end of the experiment, all mice were sacrificed under complete isoflurane anesthesia after fasting overnight. The eyeball was extirpated from each mouse to collect blood samples into tubes and were left at room temperature for 30 min to ensure full clotting, then were immediately centrifuged at 4°C, 3,500 rpm for 10 min to obtain the serum for later biochemical assays. Interscapular brown adipose, epididymal, subcutaneous, mesenteric fat pads were carefully stripped and weighed up. Intestine was removed and gently flushed with cold PBS until luminal contents appeared clear. Thereafter, it was cut into four segments, proximal jejunum, distal jejunum, ileum and colon, which were opened longitudinally. The proximal of each intestinal segment was snap-frozen in liquid nitrogen and stored at −80°C, following the distal part of each segment rolled and fixed in 4% paraformaldehyde solution for further analysis. A portion of liver, white and brown adipose tissues were also fixed with 4% paraformaldehyde for histopathological evaluation and the rest liver was kept in liquid nitrogen and then stored at −80°C refrigerator until study analysis. Fresh fecal pellets were collected from each mouse into a sterile tube for 2 days and frozen at −80°C prior to the termination of this experiment. Some of this were used for microbiota and BA analysis, some for subsequent fecal microbiota transplantation (FMT) experiment.

### Oral Glucose (OGTT) and Insulin Tolerance (ITT) Tests

Over the 14-weeks study periods, an oral glucose tolerance test (OGTT, 1 g of glucose/kg body weight), was conducted at week 12 after a 16 h fast. Blood glucose levels at 0, 15, 30, 60, 90, and 120 min after glucose administration were measured from snipped tails by Accu-chek glucometer (Bayer). In addition, insulin tolerance tests (ITT, 0.75 UI/kg body weight), at week 13, was performed after a 6 h fast. Blood glucose levels at 0, 15, 30, 60, 90, and 120 min after insulin injection were measured from snipped tails with Accu-chek glucometer (Bayer). Meanwhile, blood samples (∼30 μL) were collected from the orbital venous plexus at 0 min during ITT for fasting insulin determination. The homeostasis model assessment of insulin resistance (HOMA-IR) index was calculated using the formula: fasting insulin (*μ*UI/mL) x fasting glucose (mM)/22.5.

### Plasma Biochemistry Analysis

The serum levels of aspartate aminotransferase (AST), alanine aminotransferase (ALT), triglyceride (TG) and total cholesterol (TC) were tested on a Hitachi 7180 fully automatic biochemical analyzer (Tokyo, Japan) according to established method. Serum lipopolysaccharide (LPS) was quantified by a commercially available limulus amebocyte lysate kit (BioWhittaker, Lancaster, MA) and serum insulin was assessed using a mouse ultrasensitive enzyme-linked immunosorbent assay (ELISA) kit (Alpco, United States) following the manufacturer’s instructions.

### Histological Analysis

Four percent paraformaldehyde fixed samples including interscapular brown adipose, epididymal fat, liver tissue, and intestinal segments were embedded in paraffin after dehydration in an ethanol series. Then the paraffin-embedded samples were cut into 4 μm sections, followed by deparaffinization and hydration, and subjected to routine haematoxylin and eosin (H&E) staining. Colonic tissues were also stained with periodic acid-Schiff (PAS) according the method described by Li et al. (Li et al., 2018).

A light microscope with digital camera (Leica, Germany) was used to observe and photograph pathological changes. The degree of adipose morphology, hepatic steatosis and colon inflammation were evaluated by H&E staining. Goblet cells quantity colored with PAS in colonic gland was counted for barrier function evaluation. And 100 consecutive intact crypts were randomly selected to calculate the percentage of positive cells.

### Immunohistochemistry and Immunofluorescence

The expression of mucin 2 (MUC2), produced by goblet cells, was evaluated by immunohistochemistry. Briefly, 4 μm thick deparaffinized colon sections were first incubated with primary antibody (rabbit monoclonal anti-MUC2; Santa Cruz Biotechnology) overnight at 4°C. Then, after washing with PBS, 30 min incubation at 37°C with biotinylated anti-rabbit secondary antibody (Santa Cruz Biotechnology) was done. Next, horseradish peroxidase (HRP)-conjugated streptavidin solution was used to counterstain. The positive MUC2 staining cells were dyed brown and observed under light microscope.

For immunofluorescent staining, summarily, tissue slices (4 μm thick) were incubated at 4°C with first antibodies: anti-ZO-1 (rabbit anti mouse; Cell Signaling Technology) used in colon, and anti-FGF15 (rabbit anti mouse; Abcam) in ileum overnight. After that, 1 h secondary incubation with fluorochrome-conjugated antibody IgG H&L (anti-rabbit, Cell Signaling, Technology) was conducted at room temperature. Last, DAPI (4, 6-diamidino-2-phenylindole, blue) was used to stain cell nuclei for 1 h at 37°C. ZO-1 and FGF15 positive cells were subsequently visualized under fluorescence microscopy.

### Real-Time Quantitative PCR

Preparation of total RNA extraction from colon, ileum and liver frozen tissues were homogenized in TRIzol reagent (Invitrogen, La Jolla, CA) and purified with organic solvent. The concentration of RNA was determination on spectrophotometer Infinite M200 Pro (Tecan, Männedorf Switzerland) and then was reverse transcribed to cDNA using TIANScript RT Kit (TIANGEN, Inc. Beijing, China). After that, a quantitative real time PCR reaction mixture containing cDNA, RNase-free water, TaqMan Gene Expression Master Mix and primers (Genewiz, Inc., Beijing, China) was launched and run on StepOne Plus real time PCR instrument (Applied Biosystems, Carlsbad, CA). Relative mRNA expression of target genes, normalized to glyceraldehyde-3-phosphatedehydrogenase (GAPDH), were obtained via 2^−ΔΔCt^ method. All experimental procedures were performed strictly following manufacturer’s protocol. Primers sequences (forward and reverse) of target genes were listed in Table 1.

**TABLE 1:** Primers sequences of target genes used in real-time PCR.

Primers	Sequence
mGAPDH	Forward 5′ GGA​GAA​ACC​TGC​CAA​GTA​TG 3′
	Reverse 5′ TGG​GAG​TTG​CTG​TTG​AAG​TC 3′
mIL-1β	Forward 5′ GTG​GCT​GTG​GAG​AAG​CTG​TG 3′
	Reverse 5′ GAA​GGT​CCA​CGG​GAA​AGA​CAC 3′
mIL-6	Forward 5′ CCA​GTT​GCC​TTC​TTG​GGA​CT 3′
	Reverse 5′ GGT​CTG​TTG​GGA​GTG​GTA​TCC 3′
mTNF-*α*	Forward 5′ CTT​CTG​TCT​ACT​GAA​CTT​CGG​G 3′
	Reverse 5′ CAG​GCT​TGT​CAC​TCG​AAT​TTT​G 3′
mOccludin	Forward 5′ CGG​TAC​AGC​AGC​AAT​GGT​AA 3′
	Reverse 5′ CTC​CCC​ACC​TGT​CGT​GTA​GT 3′
mOccludin	Forward 5′ TCG​CCC​AAG​TCG​ACA​CTC​A 3′
	Reverse 5′ GCA​AAT​AGC​CAT​AGT​ACA​GTT​ACA​CAG​C 3′
mZO-1	Forward 5′ GGG​CCA​TCT​CAA​CTC​CTG​TA 3′
	Reverse 5′ AGA​AGG​GCT​GAC​GGG​TAA​AT 3′
mFXR	Forward 5′ GGA​CGG​GAT​GAG​TGT​GAA​G 3′
	Reverse 5′ TGA​ACT​TGA​GGA​AAC​GGG​AC 3′
mCYP7A1	Forward 5′ AGG​CAT​TTG​GAC​ACA​GAA​G 3′
	Reverse 5′ACA​GAT​TGG​AGG​TTT​TGC​AT 3′

### Western Blotting

Ileum tissues were lysed in ice-cold RIPA buffer with protease inhibitors (Solarbio, Beijing, China) followed by homogenization and centrifugation (12,000 g, 4°C, 15 min). The protein concentration of samples was measured by bicinchoninic acid protein assay (Thermo Scientific, Inc.) after supernatants collection. Later, protein extracts were loaded onto a 10% acrylamide gel, separated by SDS-PAGE systems, and then transferred onto PVDF membranes. The membranes were then blocked with 5% BSA (Solarbio, Beijing, China) for 1 h at room temperature, incubated with primary antibodies to FXR (rabbit anti-mouse; Abcam, Cambridge, MA, abs122163) and FGF15 (rabbit anti-mouse; Santa Cruze Biotechnology, United States, sc514647) overnight at 4°C. After the first antibody incubation, HRP-conjugated secondary antibodies were used for incubation again. The bands were visualized using a ECL Western Blotting Substrate (Solarbio, Beijing, China) with a Bio-RAD ChemiDoc XRS + Chemiluminescent Imaging System (Bio-RAD, Hercules, CA United States). The intensity of band was normalized to *β*-Actin and quantified by Image J software.

### Gut Microbiota Analysis

Total bacterial DNA from each fecal samples was extracted using improved protocol based on the manual of QIAamp Fast DNA Stool Mini Kit (Qiagen, Germany). Each 200 mg of feces, 1 ml of inhibitEX buffer and suitable amount of gleass beads were added into a sterile tube to homogenize together. The quantity of extracted bacterial DNA was determined on a NanoDrop-2000 spectrophotometer (Thermo Fisher Scientific, Waltham, MA, United States), and quality of it was controlled by 1% agarose gel electrophoresis.

Primers 341F 5' -CCTACGGGRSGCAGCAGCAG-3 ′and 806R 5' -GGACTACVVGGGTATCtaATC-3' (specific barcode was included in the primers) were used to amplify V3-V4 hypervariable region of bacterial 16S rRNA genes by PCR. PCR reactions were performed in 30 μL mixture containing 15 μL of 2 × KAPA Library Amplification ReadyMix, 1 μL of each primer (10 μM), 50 ng of template DNA and ddH2O. Negative controls consisting of empty sterile storage tubes were processed for DNA extraction, amplification using the same procedures and reagents used for the fecal samples. No amplification was detected in the negative controls.

Using the 2% agarose gel electrophoresis, the PCR products were detected. According to the manufacturer’s instructions, the PCR products was purified by AxyPrep DNA Gel Extraction Kit (Axygen Biosciences, Union City, CA, U.S.) and quantified by Qubit®2.0, and then each sample was mixed in proportion. Illumina NovaSeq (Illumina, Inc., CA, United States) was adopted for two-end sequencing. In order to acquire longer reads in high variable regions, using PANDAseq (https://github.com/neufeld/pandaseq, version 2.9) to splice.

16S sequences were controlled between 220 and 500bp, ensuring that the average Phred score of bases was no worse than 30 (Q30) and no more than 1 N. Clean reads with identical sequences were sorted by abundance and Singletons were filtered. Operational Taxonomic Units (OTUs) were clustered with 97% similarity using UPARSE (http://drive5.com/uparse/) and chimeric sequences were identified and removed using Usearch (version 7.0.1090). Each representative tags was assigned to a taxa by RDP Classifer (http://rdp.cme.msu.edu/) against the RDP database (http://rdp.cme.msu.edu/) using confidence threshold of 0.8. Becase of the great disparity between different samples, when the sample reaching sufficient sequencing depth, random leveling treatment was performed for each sample with the aim of reducing analysis bias. Sequencing depth was measured by the Alpha diversity index. OTU profiling table and alpha diversity indices were implemented through the python script of QIIME (version 1.9.1). DNA extraction, Library construction and sequencing were conducted at Realbio Genomics Institute (Shanghai, China).

### BA Analysis

Each 10 mg of freeze-dried fecal sample was placed into an Eppendorf centrifuge tube with safety claps. 200 *μ*L aliquot of acetonitrile/methanol solvent (8:2, containing 10 μL internal standards) was added to this tube. After homogenization and centrifugation (13,500 g, 4°C, 20 min), 10 μL supernatant was transferred into a tube, diluted with a 90 *μ*L mixture solvent [acetonitrile/methanol (8:2): ultrapure water = 1:1]. The supernatant was then prepared to analyze after oscillating centrifugation of extraction mixture. The six isotope internal standards were GCA-d4, TCA-d4, GDCA-d4, CA-d4, DCA-d4 and LCA-d4 at 400 nM each.

BA analysis was performed on ultra performance liquid chromatography coupled with triple quadrupole mass spectrometry (UPLC-TQMS, Waters, Milford, MA) with gradient elution at negative electrospray ionization mode. BA concentration in each sample was calculated relying on peak area and standard curve, which was set up with 23 standards at series different concentration levels. The comprehensive profiling and quantitation of the BAs were conducted at Metabo-Profile Inc. (Shanghai, China) using a protocol previously established with minor modifications (Xie et al., 2015, 2016).

### Fecal Microbiota Transplantation

Fecal samples collected from group HFD, HFDG and HFDGA were suspended in sterile phosphate-buffered saline (PBS, containing 0.05% cysteine hydrochloride, 200 mg/5 ml), which was vortexed for 3 min and rest for 2 min in ice under anaerobic environment. The supernatant was collected using Hungate anaerobic tube and stored at −80°C. Before FMT, 8-week-old C57BL/6J mice (*n* = 15) were acclimatized and received antibiotic cocktail drinking water mentioned above to eliminate intestinal origin bacteria for 1 week. After that, these recipient mice were randomly distributed into three groups: FMT-HFD group (received fecal supernatant from HFD group); FMT-HFDG group (received fecal supernatant from HFDG group) and FMT-HFDGA group (received fecal supernatant from HFDGA group). All groups were fed a normal chow diet and received sterilized water throughout this FMT experiment. Each recipient mouse was gavaged with 200 *μ*L of respective fecal supernatant each time. And the gavage performed 3 times per week for 1 month. Before the FMT experiment, fecal supernatant from the donor have been calculated on agar plate to ensure successful colonization into recipient mice. Samples of blood, liver, and intestine were also collected as described above after mice sacrifice.

### Statistical Analysis

SPSS 22.0 (SPSS, Chicago, Illinois, United States) and Origin Pro 9 software (Origin Lab Corporation, Wellesley Hills, Massachusetts, United States) were used to perform all data statistical analysis. GraphPad Prism 8.3.0 (San Diego, California, United States) was applied to generate all the bar plots. Significant differences analysis was conducted by One-way analysis of variance (ANOVA) using the Sidak test with significant criteria set to be *p* value < 0.05 for the selected pairs (NCD *vs* NCDG; NCD *vs* HFD; HFD *vs* HFDG; HFDG *vs* HFDGA) in multiple comparisons. And all the data were expressed as mean ± SEM.

## Results

### DG Prevents Weight Gain and Adiposity in HFD-Fed Mice

**FIGURE 1:**
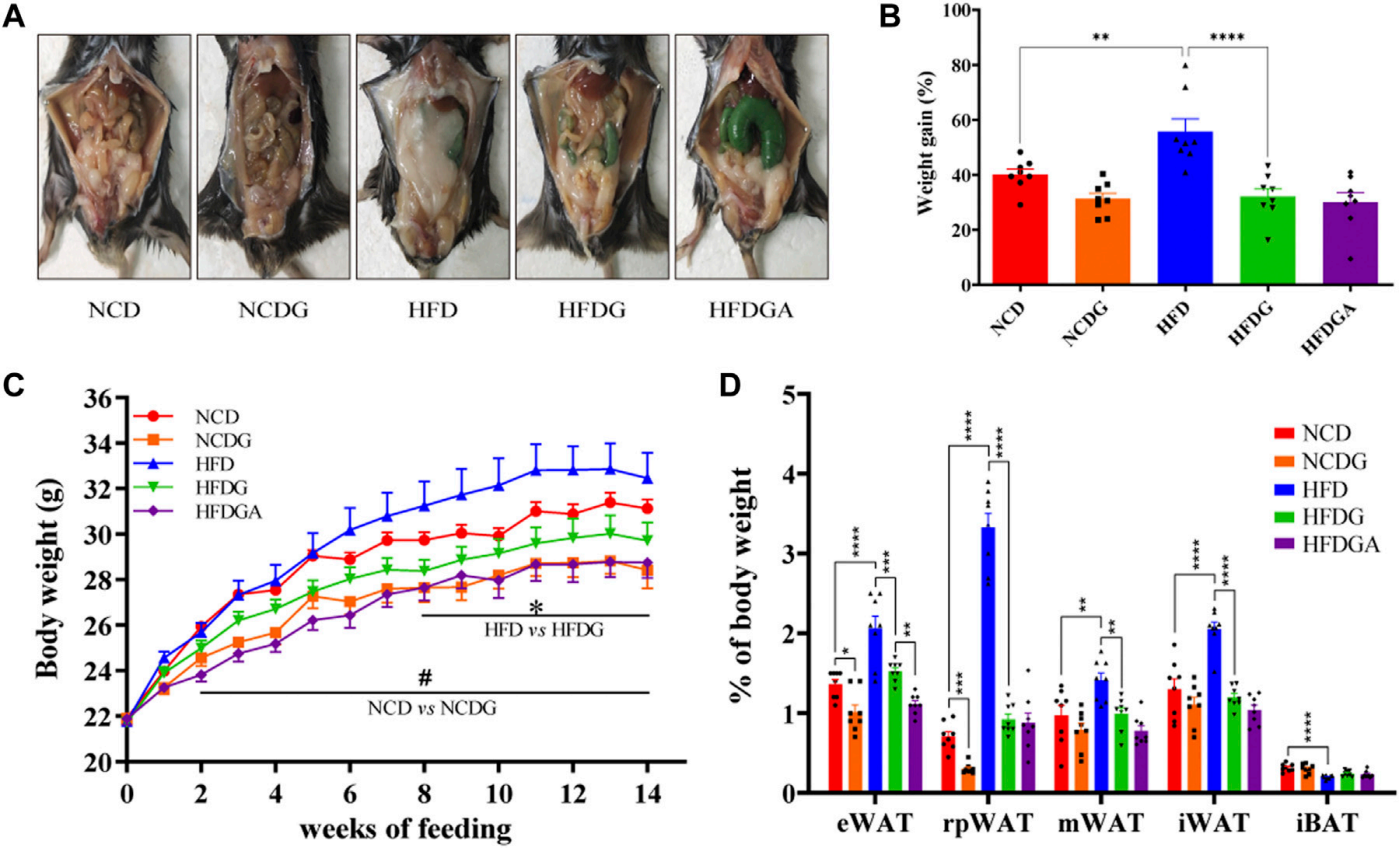
DG inhibited obesity in mice induced by high fat diet and reduced body weight in mice fed with normal chow diet. **(A)** Representative pictures of abdominal adipose in each group at 14 weeks. **(B)** Body weight gain of each group at 14 weeks. **(C)** Body weight of mice was monitored weekly for 14 weeks. **(D)** eWAT, rpWAT, mWAT and iWAT together with iBAT were collected and weighed during dissection of mice in each group. Values were denoted as mean ± SEM (*n* = 8). *p < 0.05; **p < 0.01; ***p < 0.001; ****p < 0.0001. Abbreviations: DG, diammonium glycyrrhizinate. eWAT, white adipose tissue (WAT) of the epididymis. rpWAT, WAT of the retroperitoneum. mWAT, WATof the mesentery. iWAT, WAT of the inguen. iBAT, interscapular brown adipose tissue.

Male 6-week-old C57BL/6J mice were maintained on a normal chow diet (NCD group) or high fat diet (HFD group) for 14 weeks. Concurrently, half of the mice in each of these two groups were administered with 120 mg/kg DG (NCDG and HFDG groups). After 14 weeks of DG intervention, both NCD and HFD mice displayed a significant reduction in body weight (Figures 1A–C). Meanwhile, the results (Figures 1A–C) clearly showed that HFD indeed leads to obesity, and this symptoms could be reversed to normal levels by DG supplementation. Additionally, HFD-fed mice markedly increased white adipose tissues (WAT) mass accompanied interscapular brown adipose tissue (iBAT) mass dramatically decreased compared with NCD mice (Figure 1D). Whereas supplement with DG (HFDG group) prevented all kinds of WAT accumulation, including visceral (ie, epididymal, retroperitoneal and mesenteric) and subcutaneous (ie, inguinal) in HFD mice. In contrast to the changes observed in WAT, iBAT mass was enhanced in a certain degree after the DG intervention, yet not significantly (Figure 1D). NCDG group has similar effect to those in the HFDG group versus their control mice, but only epididymal WAT (eWAT) and retroperitoneal WAT (rpWAT) were significantly less (Figure 1D), these results suggested that the weight loss effect of DG on NCD mice was mainly dependent on those two WATs. It was interesting to note that when antibiotic intervention was taken to knock out gut microbiota in HFDG mice (HFDGA group), the mice body weight in this group showed decline relative to HFDG group without statistically significant difference (Figure 1C), and this influence was mainly due to the significant reduction of eWAT (Figure 1D). This implied that combination of antibiotics could might enhance weight loss effect of DG.

### DG Improves Glucose Homeostasis and Insulin Resistance in HFD-Fed Mice

**FIGURE 2:**
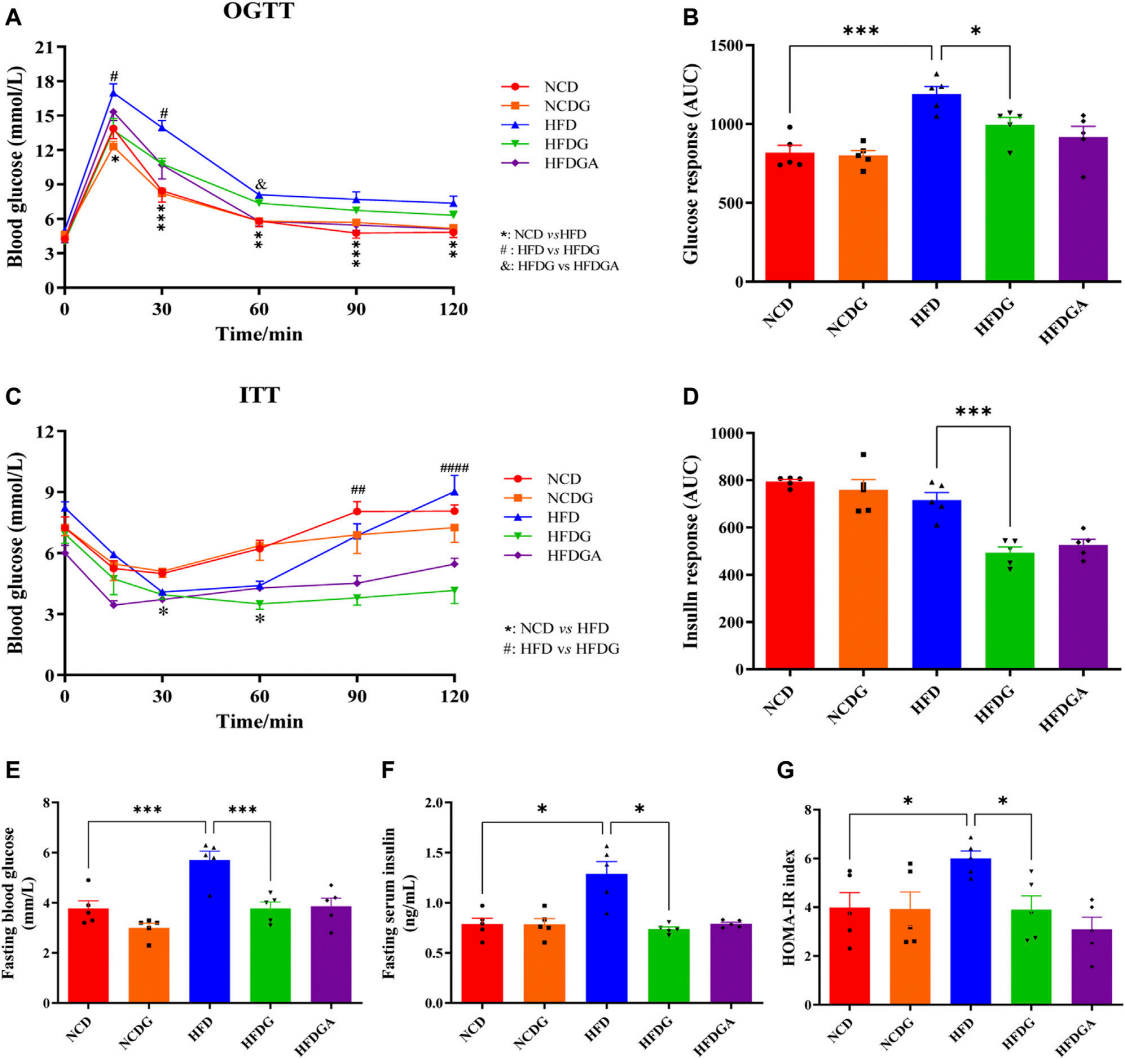
DG reduced glucose intolerance and insulin resistance in mice fed with high fat diet. **(A, C)** OGTT and ITT in each group, respectively. **(B, D)** The calculated AUC for OGTT and ITT, respectively. **(E, F)** Fasting blood-glucose and insulin, respectively. **(G)** HOMA-IR based on the formula. Values were denoted as mean ± SEM (*n* = 5). *p < 0.05; **p < 0.01; ***p < 0.001. Abbreviations: DG, diammonium glycyrrhizinate. OGTT, oral glucose tolerance test. ITT, insulin tolerance test. AUC, area under the curve. HOMA-IR, homeostasis model assessment of insulin resistance.

We next investigated the impact of DG on glucose homeostasis and insulin sensitivity in HFD mice. HFD strongly elevated the glucose levels at various time points in the oral glucose tolerance test (OGTT) compared to NCD, and DG significantly reduced the blood glucose levels within 30 min after taking glucose orally, which meant DG accelerated glucose clearance (Figure 2A). What was interesting observation in Figure 2A was that a slight reduction of blood glucose levels existed in HFGDA group than HFDG with no significance. DG also could improve the HFD mice insulin sensitivity in insulin tolerance test (ITT), especially 60 min after insulin injection (Figure 2C). Consistent with this, the mice in HFDG group displayed dramatically lower area under the curve (AUC) values in OGTT (Figure 2B) and ITT (Figure 2D) versus mice in HFD group. Higher fasting plasma glucose (Figure 2E), insulin (Figure 2F), and HOMA-IR (Figure 2G) induced by HFD were remarkably prevented by HFDG.

### DG Ameliorates Liver Steatosis and Related Indexes in HFD-Fed Mice

**FIGURE 3:**
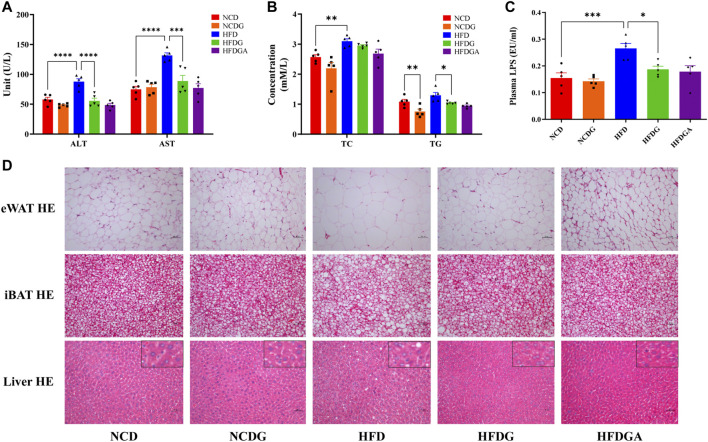
DG improved liver and tissue steatosis in mice fed with high fat diet. **(A)** Serum ALT and AST in each group. **(B)** Serum TC and TG in each group. **(C)** Serum level of LPS in each group. **(D)** Pathological examination of eWAT, iBAT and liver by H&E staining in each group (scale bar: 50 *μ*m). Values were denoted as mean ± SEM (*n* = 5). *p < 0.05; **p < 0.01; ***p < 0.001; ****p < 0.0001. Abbreviations: DG, diammonium glycyrrhizinate. ALT, alanine aminotransferase. AST, aspartate aminotransferase. TC, total cholesterol. TG, total triglyceride. LPS, lipopolysaccharide. eWAT, white adipose tissue of the epididymis. iBAT, interscapular brown adipose tissue.

After 14 weeks, the plasma alanine aminotransferase (ALT) and aspartate aminotransferase (AST) levels were significantly higher in HFD fed mice than those in NCD group, which were distinctly dropped to normal level by HFDG (Figure 3A). On the other hand, feeding on a high-fat diet resulted in a significant increase in serum total cholesterol (TC) and a slight rise in total triglyceride (TG). However, after DG intervention, these indicators showed a more or less decreasing trend, and obviously decreased in TG. This similar phenomenon was also observed in the NCD group (Figure 3B). LPS is a chemical component peculiar to the outer layer of Gram-negative bacteria. As a common endotoxin, it can synthesize and release a variety of cytokines and inflammatory mediators through cell signal transduction system *in vivo*, leading to a series of reactions in the body (Cani et al., 2007; Boutagy et al., 2016). In this study, serum LPS level was sharply raised in HFD mice, and supplementation with DG prominently inhibited the increase of it (Figure 3C). We then examined the adipocytic morphology, BAT metabolism, and hepatic histology by H&E staining. As shown in Figure 3D, lager eWAT adipocyte cells, severer iBAT whitening, and hepatocyte microvesicular steatosis were exhibited in HFD group, and when it was supplied with DG, each of these changes has been improved (Figure 3D). In addition, other groups were about the same as NCD group.

### DG Enhances Intestinal Barrier and Reduces Intestinal Low-Grade Inflammation in HFD-Fed Mice

**FIGURE 4:**
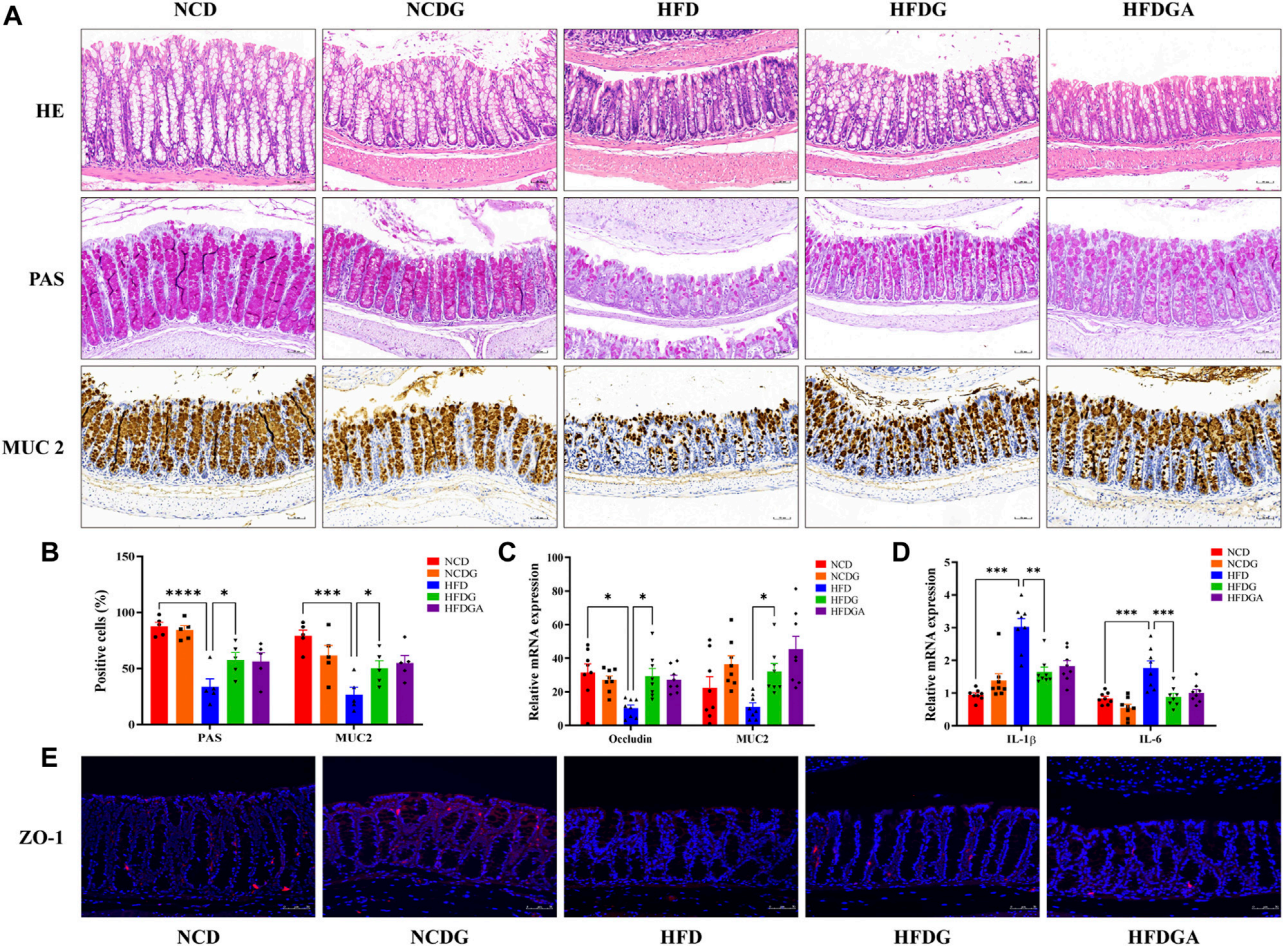
DG ameliorated intestinal barrier impairment and low-grade inflammationin of colon in high fat diet mice. **(A)** H&E staining, PAS staining for goblet cells, and Immunohistochemistry staining for MUC2 (scale bar: 50 μm). **(B)** The number of positive cells of PAS and MUC2 staining in each colonic crypt (*n* = 5). **(C)** Relative mRNA expression of colonic occludin and MUC2 (*n* = 8). **(D)** Relative mRNA expression of colonic IL-1β and IL-6 (*n* = 8). **(E)** Immunofluorescence staining for ZO-1 distribution of colon tissues (red, scale bar: 50 μm). Values were denoted as mean ± SEM. *p < 0.05; **p < 0.01; ***p < 0.001; ****p < 0.0001. Abbreviations: DG, diammonium glycyrrhizinate. MUC2, mucin 2. ZO-1, zonula occludens 1.

Intestinal mucosal barrier could effectively prevent harmful substances like bacteria and toxins in the gut from entering other tissues to protect host from invasion, and the intestinal mucous layer was mostly composed of goblet cells and its mucin product (Liévin-Le Moal and Servin, 2006). In this study, colonic positive cells number in these two substances stained by PAS and MUC2 respectively were considerably decreased in HFD group relative to NCD group, but HFDG group resulted in a substantial elevation (Figures 4A,B). Similarity with expression of MUC2 positive cells, the mRNA level of it was enhanced as well (Figure 4C). Tight junctions (TJ) between intestinal epithelial cells were important structural basis for gut mechanical barrier. Occludin and zonula occludens 1 (ZO-1) were representative TJ, changes of occludin mRNA expression resembled to PAS staining (Figure 4C). At the same time, as visualized by immunofluorescence (Figure 4E), comparison with group NCD, HFDG alleviated the remarkable decreasing of ZO-1in colonic membrane from HFD group. It is widely believed that damaged intestinal barrier tended to induce inflammation (Halfvarson et al., 2017; Franzosa et al., 2019), thus we detected the proinflammatory cytokine including interleukin-1beta (IL-1β) and interleukin-6 (IL-6), and we found that both of them were significantly raised in HFD colon tissue versus NCD, which were adjusted to near NCD by HFDG (Figure 4D). Meanwhile, no apparent colonic microscopic inflammation by H&E staining was observed in this experiment (Figure 4A). Besides, accumulating data in this part also suggested that DG unable to make significant influence on the NCD group, and HFDGA has no difference with HFDG, either in terms of intestinal barrier or inflammation.

### DG Inhibits Ileal FXR-FGF15 Signaling to Influence Hepatic BA Synthesis in HFD-Fed Mice

**FIGURE 5:**
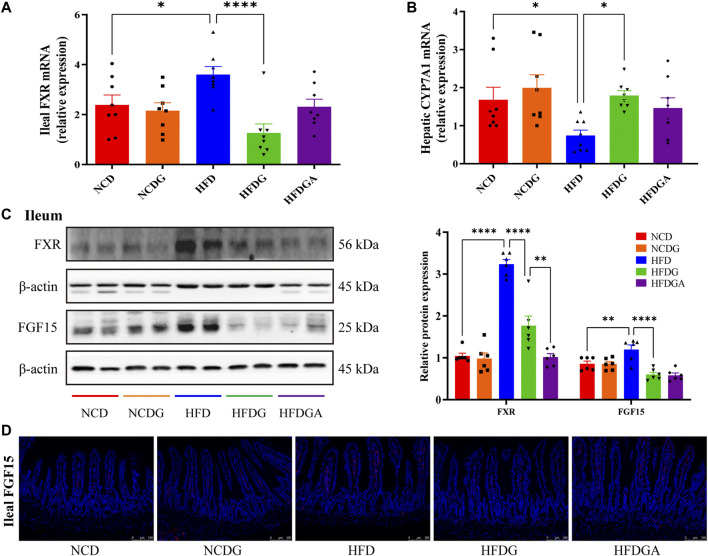
DG suppressed ileal FXR-FGF15 metabolic signaling to impact rate-limiting enzyme of hepatic bile acids (BAs) biosynthesis in high fat diet mice. **(A)** Relative mRNA expression of ileal FXR (*n* = 8). **(B)** Relative mRNA expression of liver BA synthase CYP7A1 (*n* = 8). **(C)** Relative protein expression levels of FXR and FGF15 in ileum (*n* = 6). **(D)** Immunofluorescence staining of ileal FGF15 (red, scale bar: 100 μm). Values were denoted as mean ± SEM. *p < 0.05; **p < 0.01; ***p < 0.001; ****p < 0.0001. Abbreviations: DG, diammonium glycyrrhizinate. FXR, farnesoid X-activated receptor. FGF15, fibroblast growth factor 15.

FXR, a ligand-activated nuclear receptor, which has been found to play critical roles in metabolic diseases like obesity due to its function of regulating bile acid metabolism. Meanwhile, the activation of intestinal FXR could induce the expression of FGF15 in mice (Jia et al., 2018). So both mRNA expression level of FXR and relative protein levels of FXR and FGF15 at the distal ileum of each group were investigated. As a result, FXR mRNA as well as protein expression levels were noticeably increased in HFD relative to NCD, and these tendencies were strongly inhibited at protein level and even lower at mRNA level than NCD by HFDG group (Figures 5A,C). On the other hand, similar to FXR expression, ileal FGF15 protein expression was also remarkably raised in HFD group, and was significantly down-regulated by HFDG as well (Figure 5C). Immunofluorescence of FGF15 in ileum further confirmed this observation (Figure 5D). When FGF15 was released into the portal vein, it will suppress BA synthetic rate-limiting enzymes transcription through binding FGFR4 in liver (Jia et al., 2018). Based on this, what we continually found was that the mRNA expression levels for the hepatic BA synthetic genes, CYP7A1 was mostly decreased in HFD compared with NCD, while HFDG could relieve this suppression (Figure 5B). Here, there were no significant differences existed in NCDG and HFDGA groups relative to their controls.

### DG Modulates the Composition of the Gut Microbiota and Reduced BSH Enriched Bacteria in HFD-Fed Mice

**FIGURE 6:**
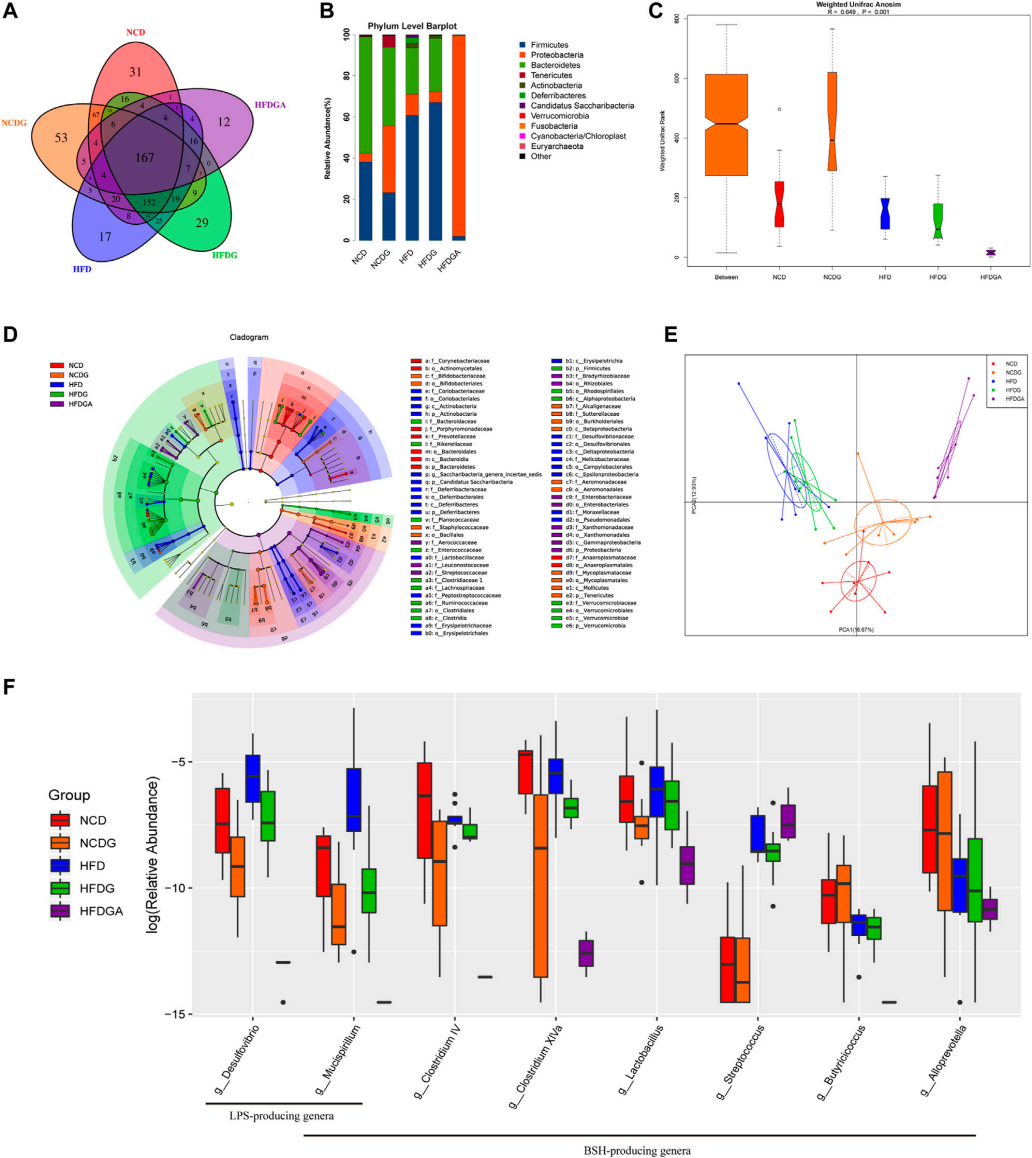
DG changed gut microbiota composition and diminished BSH enriched microbes in high fat diet mice. **(A)** OTU Venn diagram between 5 groups. **(B)** Relative abundance of gut microbiota at phylum level. **(C)** Weighted Unifrac Anosim analysis between 5 groups. **(D)** Differential analysis in the cladogram by LEfSe. **(E)** PCA results of different bacterial taxa at the genus level. **(F)** Relative abundance for specific bacterial genera of each group. *n* = 8 each group. Abbreviations: DG, diammonium glycyrrhizinate. BSH, bile-salt hydrolase. OTU, operational taxonomic unit. LEfSe, linear discriminant analysis effect size. PCA, principal components analysis.

In this section, we performed 16S rRNA gene sequences to determine the overall structural changes of gut microbiota in each group microbial samples. Venn diagram was used to evaluate the similarity and consistency of the over lapping operational taxonomic units (OUTs) of samples. As Figure 6A shown, 167 OTUs were shared in all groups. Meanwhile, 31, 53, 17, 29, 12 OTUs were respectively unique to NCD, NCDG, HFD, HFDG and HFDGA group. Which indicated DG treatment added the exclusive OTUs in NCDG and HFDG groups relative to their controls. Then, the microbiota community structures were investigated. The five dominant abundant bacteria at phylum level, namely *Firmicutes*, *Proteobacteria*, *Bacteroidetes*, *Tenericutes*, and *Actinobacteria*, were discovered in various quantities among NCD, NCDG, HFD and HFDG groups (Figure 6B). *Firmicutes* and *Proteobacteria* were still mainly present after antibiotics were given to eliminate most of the original bacteria in HFDGA group (Figure 6B). Analysis of similarity (ANOSIM) on unweighted UniFrac distances was applied to manifest the beta diversity of the five groups, and ANOSIM exhibited a significant difference between these groups (Figure 6C).

Next, we utilized linear discriminant analysis effect size (LEfSe) to estimate the influence of the abundance of each component (species) on the difference effect, and we found that *p_Actinobacteria.c_Actinobacteria.o_Coriobacteriales.f_Coriobacteriaceae*, *p_Deferribacteres.c_Deferribacteres.o_Deferribacterales.f_Deferribacteraceae*, *c_Erysipelotrichia.o_Erysipelotrichales.f_Erysipelotrichaceae*, *c_Deltaproteobacteria.o_Desulfovibrionales.f_Desulfovibrionaceae* and *c_Epsilonproteobacteria.o_Campylobacterales.f_Helicobacteraceae* had a significant effect on HFD group; *p_Verrucomicrobia.c_Verrucomicrobiae.o_Verrucomicrobiales.f_*V*Verrucomicrobiaceae* had a significant influence in HFDG group; *p_Tenericutes.c_Mollicutes.o_Mycoplasmatales.f_Mycoplasmataceae* had an obvious effect on NCDG group; and *p_Tenericutes.c_Mollicutes.o_Anaeroplasmatales.f_Anaeroplasmatacea* had a remarkable effect on NCD group (Figure 6D). The kruskal. test function of the stats package of R language was used to perform a significant difference analysis between all groups to find out the species that had a significant influence on the division between groups. A total of 60 genera with significant differences among different groups were screened by rank sum test. Figure 6E showed principal components analysis (PCA) result of different species in all groups at genus level, and it revealed distinct clustering of microbiota community structure for each experimental group. Notable changes were induced by both HFD and DG intervention groups. The microbes in HFD and HFDG were more closely clustered, again, NCD and NCDG also kept close distance, while group HFDGA located far from other groups on account of removing much of the microbes. Collectively, these results illustrated that DG caused changes in the gut microbiota indeed, together, use of antibiotics drinking water also did have hugely effect on microbes in HFD mice.

### DG Changed Fecal Composition of BAs Pool and Raised Proportion of Conjugated BAs in HFD-Fed Mice

**FIGURE 7:**
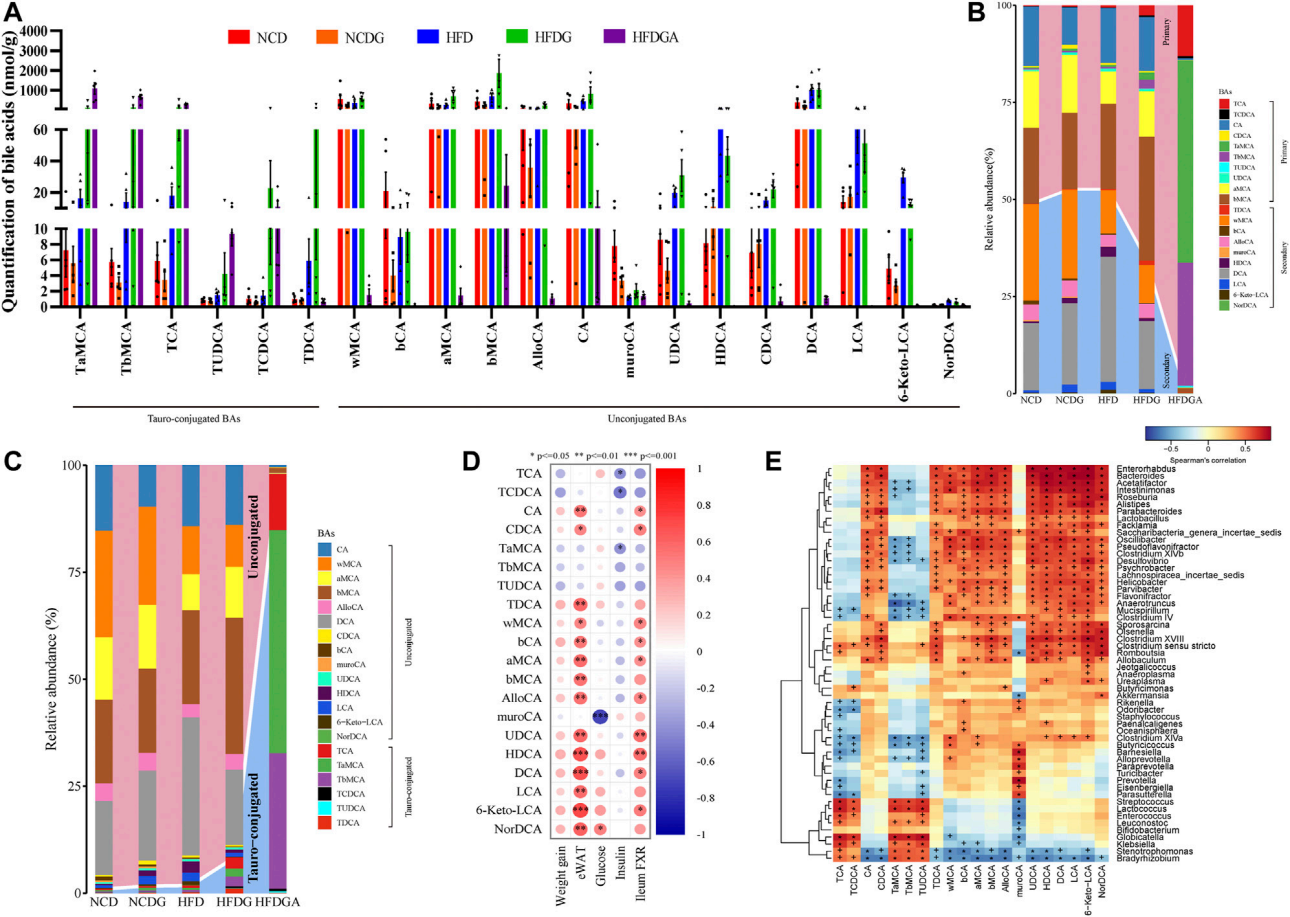
DG altered BA profiles and increased proportion of conjugated BAs in high fat diet mice. **(A)** Fecal levels of differential BAs. **(B-C)** Changes in differential BAs composition. **(D)** Spearman correlation analysis between differential BAs and conventional index including weight gain, eWAT, fasting glucose, fasting insulin, and ileum FXR. **(E)** Heat map of spearman correlation between BAs and gut microbiota at genus level identified with significant difference in fecal samples of each mice. + *p* < 0.05; *p < 0.01. Values were denoted as mean ± SEM (*n* = 5). *p < 0.05; **p < 0.01; ***p < 0.001. Abbreviations: DG, diammonium glycyrrhizinate. BAs, bile acids. eWAT, white adipose tissue of the epididymis. FXR, farnesoid X-activated receptor.

As stated in the above result, intestinal FXR signaling was involved in regulating bile acid metabolism. Since DG has been found to markedly diminish the up-regulation of the FXR-FGF15 signaling pathway induced by HFD, but whether DG could alter the profile of BAs was still unknown. Accordingly, this section in the study was concerned with this. Ultra-performance liquid chromatography tandem mass spectrometer (UPLC-MS/MS) was used to determinate BAs in the feces of each group. And one-dimensional ANOVA test or Kruskal-Wallis (K-W test) test was selected according to the normality and homogeneity of variance of the data to obtain the differential metabolites between multiple groups. A total of 23 BA species were assayed and 20 of them were screened as potential biomakers under the set conditions (Figure 7A). The specific results revealed that a rising trend in tauro α-muricholate (TaMCA), tauro β-muricholate (TbMCA), taurocholic acid (TCA), tauro deoxycholate (TDCA), β-muricholic acid (bMCA), cholic acid (CA), ursodeoxycholic acid (UDCA), α-hyodeoxycholic acid (HDCA), chenodeoxycholic acid (CDCA), deoxycholic acid (DCA), lithocholic acid (LCA) and 6-ketolithocholic acid (6-keto-LCA) as well as a decreasing trend in ω-muricholic acid (wMCA), 3β-cholic acid (bCA), α-muricholic acid (aMCA) and murocholic acid (muroCA) were detected on HFD group versus NCD group. Tauro-conjugated BAs including TaMCA, TbMCA, TCA, tauroursodeoxycholic acid (TUDCA), taurochenodeoxycholate (TCDCA) and TDCA presented an upward trend in HFDG group relative to HFD. And a rising tendency on wMCA, aMCA, bMCA, allocholic acid (AlloCA), CA, UDCA and CDCA followed a clear declining tendency on HDCA, LCA and 6-keto-LCA were also examined in HFDG group compared to HFD group. There, relative to NCD group, wMCA, bCA, aMCA, bMCA, AlloCA and CA, exhibited a downward trend in NCDG group, the rest of BAs were no clear difference. Concurrently, after antibiotic intervention, the majority of BAs were substantially dropped or even to zero in HFDGA group, however, Tauro-conjugated BAs such as TaMCA, TbMCA, TCA, TUDCA and TCDCA were still existed in mice body, especially the first four BAs were instead greatly augmented. In general, DG altered the composition of the BA pool in HFD-fed mice. Even though the ratios of primary BAs (PBAs) to secondary BAs (SBAs) (PBA/SBA ratio) in group NCD, NCDG and HFD changed a little (Figure 7B), the PBA/SBA ratio as well as the tauro-conjugated BAs to unconjugated BAs (ConBA/UnconBA ratio) were increased after DG treatment, and also there were an overwhelming increase either in PBA/SBA ratio or in ConBA/UnconBA ratio in HFDGA group (Figures 7B,C). A variety of transporters in liver and intestine are involved in the enterohepatic circulation of BAs and thereafter affect host physiology and pathology (Schneider et al., 2018). In this case, we simply examined the mRNA expression changes of some BA transporters. The results (Supplementary Figures S1B–E) revealed that except for OSTα, where was no changes between groups in ileum, DG intervention exhibited ability that an increasing tendency in mRNA expressions of hepatic BSEP, ileal ASBT and OSTβ in HFD mice. and the intake of DG also raised the ileal mRNA level of OSTβ in NCD. When combined with antibiotics treatment (HFDGA), this rising trend was even more pronounced relative to HFDG group.

We next performed analysis based on spearman correlation between BAs mentioned above and other metabolic parameters to identify the dominant BAs responsible for adjusting metabolism. And the correlation analysis showed that most tauro-conjugated BAs like TCA, TCDCA, TaMCA, TbMCA and TUDCA were negatively correlated with levels of body weight, insulin and ileum FXR (Figure 7D). Therefore, tauro-conjugated BAs might play a beneficial roles in preventing obesity induced by HFD in this survey.

### DG Reduced BSH Enriched Bacteria and LPS-Producing Bacteria in HFD-Fed Mice

In order to look for the relationship between gut microbiota changes and various BAs, we made a further correlation analysis tried to explain the changes in conjugated BAs investigated previously (Figure 7E). Surprisingly, we screened numbers of bacteria clearly associated with BAs as expected. We found that genera like *Mucispirillum*, *Clostridium IV*, *Clostridium XlVa*, *Butyricicoccus* and *Alloprevotella* showed a significant negative correlation with tauro-conjugated BAs. Most of these microbial genera happened to possess a common function forming the Bile salt dehydrogenase (BSH) enzymes which could generate unconjugated BAs via deconjugating taurine-conjugated BAs in mice (Ridlon et al., 2006; Chiang, 2009; Wahlström et al., 2016; Jia et al., 2018). Besides, genera of *Lactobacillus* and *Streptococcus* were also found with BSH activity (Huang et al., 2019). These genera relevant with BSH enzymes were exhibited by the boxplot to see in detail how they changed in each group (Figure 6F). As we saw, antibiotic use in HFDGA group almost eliminated these genera, and DG treatment down-regulated the abundances of these BSH-producing genera relative to their control groups more or less. Here, *Desulfovibrio* and *Mucispirillum*, as LPS-producing genera (Kim et al., 2012; Zhang et al., 2020), were similarly reduced after DG intervention with respect to NCD and HFD groups (Figure 6F).

### Gut Microbiota From DG Treatment Relieved Intestinal Barrier Impairment

**FIGURE 8:**
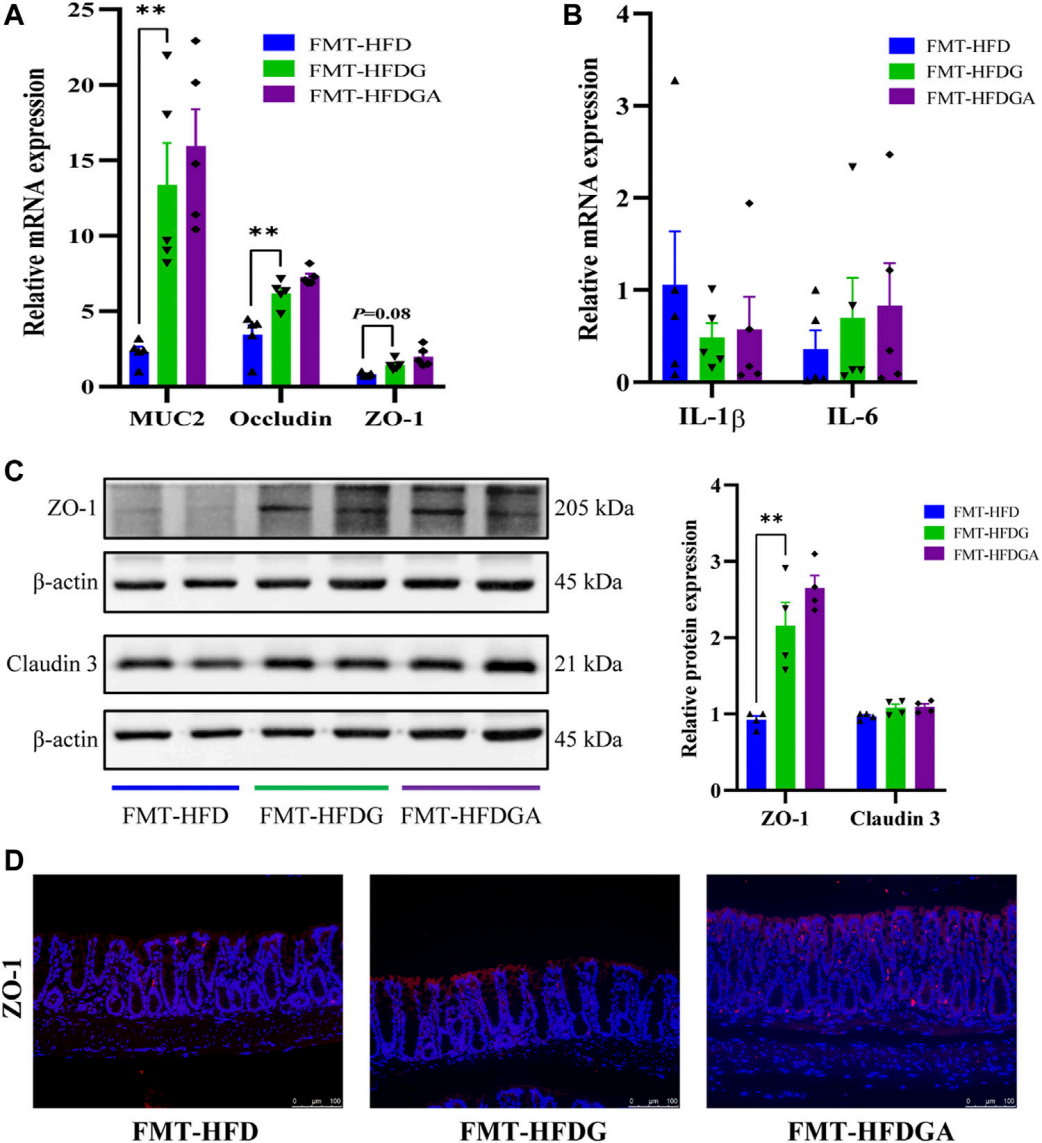
Gut microbiota from DG treatment relieved intestinal barrier impairment. **(A)** Relative mRNA expression of colonic MUC2, occludin and ZO-1 of recipient mice in FMT experiment (*n* = 5). **(B)** Relative mRNA expression of colonic IL-1β, IL-6 and TNF-α of recipient mice in FMT experiment (*n* = 5). **(C)** Relative protein expression of colonic ZO-1 and claudin three of recipient mice in FMT experiment (*n* = 4). **(D)** Immunofluorescence staining for ZO-1 distribution in colon tissues (red, scale bar: 100 μm) of recipient mice in FMT experiment. Values were denoted as mean ± SEM. **p < 0.01. Abbreviations: DG, diammonium glycyrrhizinate. HFD, high fat diet. MUC2, mucin 2. ZO-1, zonula occludens 1. FMT, fecal microbiota transplantation. IL-1β, interleukin-1beta. IL-6, interleukin-6. TNF-α, tumor necrosis factor-α.

As what referred before, high-fat diet could impair intestinal barrier function and promote intestinal low-grade inflammation in mice, and DG treatment attenuated these adverse effect. We carried out fecal microbiota transplantation (FMT) experiment to confirm again whether microbiota could affect intestinal barrier function and inflammation. As expected, the relative lower mRNA expression levels of MUC2, occludin and ZO-1 were dramatically observed in the FMT-HFD group (gavaged with fecal samples from the HFD group) compared to group FMT-HFDG (gavaged with fecal samples from the HFDG group) (Figure 8A). Whereas no difference between group FMT-HFDG and FMT-HFDGA was found in these indicators. Besides, significant lower protein expression of ZO-1 was also found in FMT-HFD relative to FMT-HFDG (Figure 8C). Also the immunofluorescence of ZO-1 in colon reaffirmed this phenomenon (Figure 8D). The relative protein levels of claudin 3, one of TJ in intestinal epithelial cells, was no difference in each group (Figure 8C). In addition, we found no significant difference in intestinal proinflammatory cytokine such as IL-1β, IL-6 and tumor necrosis factor-α (TNF-α) between groups (Figure 8B). So we concluded that unlike the intestinal barrier damage caused by gut microbiota derived from HFD, gut microbiota from DG treatment could not lead to intestinal barrier impairment in NCD mice.

## Discussion

We have observed the fact that daily treatment of HFD-fed mice with DG could prevent diet-induced obesity and alleviate metabolic syndrome. While improving metabolic phenotype, DG was further found to bring gut microbiota changes, BA profile alteration as well as intestinal barrier enhancement in obesity mice. These results reconfirmed weight loss effects of DG in previous reports (Madak-Erdogan et al., 2016; Li et al., 2018; Liou et al., 2019; Qiu et al., 2019). However, most of those reports only focus on an association between DG resistance to obesity and gut microbiota without elucidating specific functional pathway, our study sheds light on some key mechanisms behind the beneficial effects of DG therapy.

BSH, enriched in intestinal microbes, catalyzes hydrolysis reaction that glycine and taurine in conjugated BAs are removed to generate unconjugated BAs, which subsequently produce a diverse array of secondary BAs through undergoing various microbial biotransformations like dehydroxylation, epimerization and oxidation of hydroxyl groups (Gonzalez et al., 2016; Wahlström et al., 2016). The BA deconjugation process is dominated by bacteria with BSH activity, which was mainly attributed to *Lactobacillus*, *Bifidobacterium* and *Clostridium* (Tannock et al., 1989; Tanaka et al., 1999; Begley et al., 2006). A report from an earlier study showed that the antioxidant tempol decreased the relative abundance of BSH-producing *Lactobacillus*, leading to changes in BA composition, and ultimately improved food-borne obesity (Sayin et al., 2013). Also inhibition of BSH-producing microbes by oral administration of ampicillin increased the levels of tauro-conjugated BAs (TCA and T-βMCA), suppressed ileal FXR expression and resulted in obesity resistance (Kuribayashi et al., 2012). As mentioned in the similar report, in our study, we found that the genera of *Mucispirillum*, *Clostridium IV*, *Clostridium XlVa*, *Butyricicoccus* and *Alloprevotella* were strongly negative correlated with conjugated BAs level such as TCA, TCDCA, TαMCA, TβMCA and TUDCA. Some of these genera have been proved to be the active bacteria of BSH, and DG intervention decreased the relative abundances of these genera, especially reduced the BSH-producing *Clostridium IV*, and *Clostridium XlVa* genera in mice. The content of tauro-conjugated BAs was therefore upregulated, and finally reduced the body weight of NCD mice as well as inhibited obesity of HFD mice. Additionally, these changes were more pronounced in antibiotic-treated mice (HFDGA), and this phenomenon was both in harmony with the original purpose of using antibiotics, namely microbiota-elimination to simulate germ-free mice, again it corroborated the findings of previous work. (Li et al., 2013; Sayin et al., 2013). This may explain the fact that combination with antibiotics treatment could slightly enhance weight loss effect of DG in high fat diet mice. In detail, it may be due to that changes in gut microbiota caused by antibiotics treatment alone can already achieve a certain obesity resistance, and DG may also present an “antibiotic-like effect” on weight loss. Logically, when mice subjected to HFD and treated simultaneously with DG and antibiotics (HFDGA), it performed greater weight loss in obese mice than treated with DG alone.

BAs, produced in the liver from cholesterol and metabolized in the intestine by the gut microbiota, can activate corresponding nuclear receptors (FXR and TGR5) by acting as endogenous signaling molecules. The activation of these receptors alter gene expression in multiple tissues, thereby leading to changes in bile acid metabolism, glucose homeostasis, lipid metabolism, energy expenditure (Chávez-Talavera et al., 2017; Shapiro et al., 2018; Krautkramer et al., 2021). Gut microbiota is closely related to BA metabolism, and this close relationship is mainly regulated by ileal FXR-FGF15 signaling axis. Once changes in ileal FXR-FGF15 axis, BA-synthetic rate-limiting enzyme CYP7A1 in liver will be affected (Wahlström et al., 2016). Different BAs activate FXR with different degrees. The most effective ligand for FXR is CDCA, followed by CA, DCA and LCA (Makishima et al., 1999). UDCA does not activate FXR, instead, it inhibits FXR activation (Mueller et al., 2015). While, T-αMCA and T-βMCA are characterized as naturally FXR antagonist (Sayin et al., 2013). Recently, an experiment showed that HFD fed mice treated with orally administration of conjugated BA (TCDCA or TUDCA) finally exhibited decreased expression of ileal FXR-FGF15 signaling and accelerated BA synthesis (Huang et al., 2019); another study indicated that the reduction of ileal FXR signaling induced by ampicillin was restored by oral administration of uncoupled CA rather than coupled TCA (Kuribayashi et al., 2012). Both of these two researches seem to emphasize that conjugated BAs may be antagonistic to ileal FXR. These findings were further confirmed in our experiment. HFD mice with DG treatment sharply elevated levels of conjugated BAs. Particularly, we also found DG increased the ratio of FXR antagonists to agonists in BAs (Supplementary Figure S1A), in parallel, similar appearance was obviously seen in microbiota-depleted mice, followed by inhibition of ileal FXR-FGF15 axis, then enhances mRNA expression of hepatic BA synthetic gene CYP7A1 along with BA pool changed and liver cholesterol more consumption, ultimately results in decreased levels of serum TC and TG.

The role of FXR is complex, depending on the type of tissue and environmental factors. Both FXR agonists and antagonists have been reported to have health benefits in different contexts. There are studies suggested that activation of FXR was involved in the occurrence and development of nonalcoholic fatty liver disease by regulating bile acid homeostasis, lipid metabolism, inflammatory response, liver fibrosis, liver regeneration and other links (Xi and Li, 2020). Research has showed that hepatic FXR activation mainly interferes with hepatic fatty acid uptake and inhibits lipid synthesis in a small heterodimer partner (SHP) ligand dependent manner, thus improving liver fat accumulation (Jadhav et al., 2018). Meanwhile, it has also been found activation of intestinal FXR induces the expression of FGF15/19, which leads to SHP phosphorylation, thus recruiting DNA methyltransferase 3A to bind to lipid-producing genes, inhibiting hepatic steatosis (Kim et al., 2020). In view of this, multiple FXR agonists have been developed and are in various clinical trial stages for the treatment of Nonalcoholic steatohepatitis (Kremoser, 2021). Although FXR activation has a protective effect on steatosis, some other voices also revealed that intestinal FXR promotes diet-induced obesity and steatosis (Wang et al., 2018; Krautkramer et al., 2021). With the in-depth research of intestinal FXR, it has been found not only play an important role in maintaining BAs homeostasis and regulating glucose and lipid metabolism, but also play a protective role in the intestinal tract by reducing intestinal inflammation and strengthening intestinal mucosal barrier. One literature showed that inhibition of intestinal FXR or FXR deficiency could promote glucagon-likepeptide-1 (GLP-1) secretion thus improving obesity-induced glucose intolerance and insulin resistance (Sun et al., 2018). Another previous report presented that the dissociation of conjugated BAs by gut microbiota activated intestinal FXR to produce ceramides, which promote the synthesis and accumulation of liver fat and suppress the expression of heat related genes in beige fat cells (Jiang et al., 2015b). Additionally, a recent paper provided evidence that palmatine-treated rats reduced plasma LPS content and increased intestinal permeability via down-regulating the ileal FXR expression (Ning et al., 2020). Consistent with those studies mentioned later, we observed that when DG was supplemented, HFD mice were characterized by several obesity-related metabolic improvements including lower serum glucose and insulin levels, increased insulin sensitivity, few hepatic steatosis and resistance to weight gain by ileal FXR-FGF15 signaling inhibition. On the other hand, decreased level of serum LPS and higher intestinal barrier were also identified in HFD mice with DG treatment.

Studies have reported that gut microbiota dysbiosis can cause intestinal barrier disruption as well as host immune and energy metabolism disorders (Gomes et al., 2018; Allam-Ndoul et al., 2020; Tilg et al., 2020). Damaged intestinal barrier cause intestinal wall leakage leading to LPS entering systemic circulation, inducing chronic inflammation, and contributing to metabolic disorders like obesity and insulin resistance by multiple pathways (Cox and Blaser, 2013; Ghosh et al., 2018; Gomes et al., 2018). Mutually, when gut microbiota is maladjusted, especially increased in Gram-negative bacteria, where LPS in the cell wall is released, which can in turn destroy and penetrate intestinal mucosa (Guo et al., 2015). In current study, our results reflected that high fat diet drove to gut microbiota changes and barrier destruction, microbiota analysis further manifested levels of some LPS-producing bacterial genera such as *Desulfovibrio* and *Mucispirillum* were more existed in HFD, which agree with characteristics of obesity in previous reports (Kim et al., 2012; Guo et al., 2018). However, adding with DG reversed this trend, following by colonic inflammation reduction. Combination with our FMT experiment, we might assume that HFD could indeed destroy intestinal barrier through regulating the gut microbiota, mainly due to upregulation of LPS-producing genera of *Desulfovibrio* and *Mucispirillum*. And DG intervention reduced these two LPS-producing genera to prevent the gut barrier damage caused by HFD.

**FIGURE 9:**
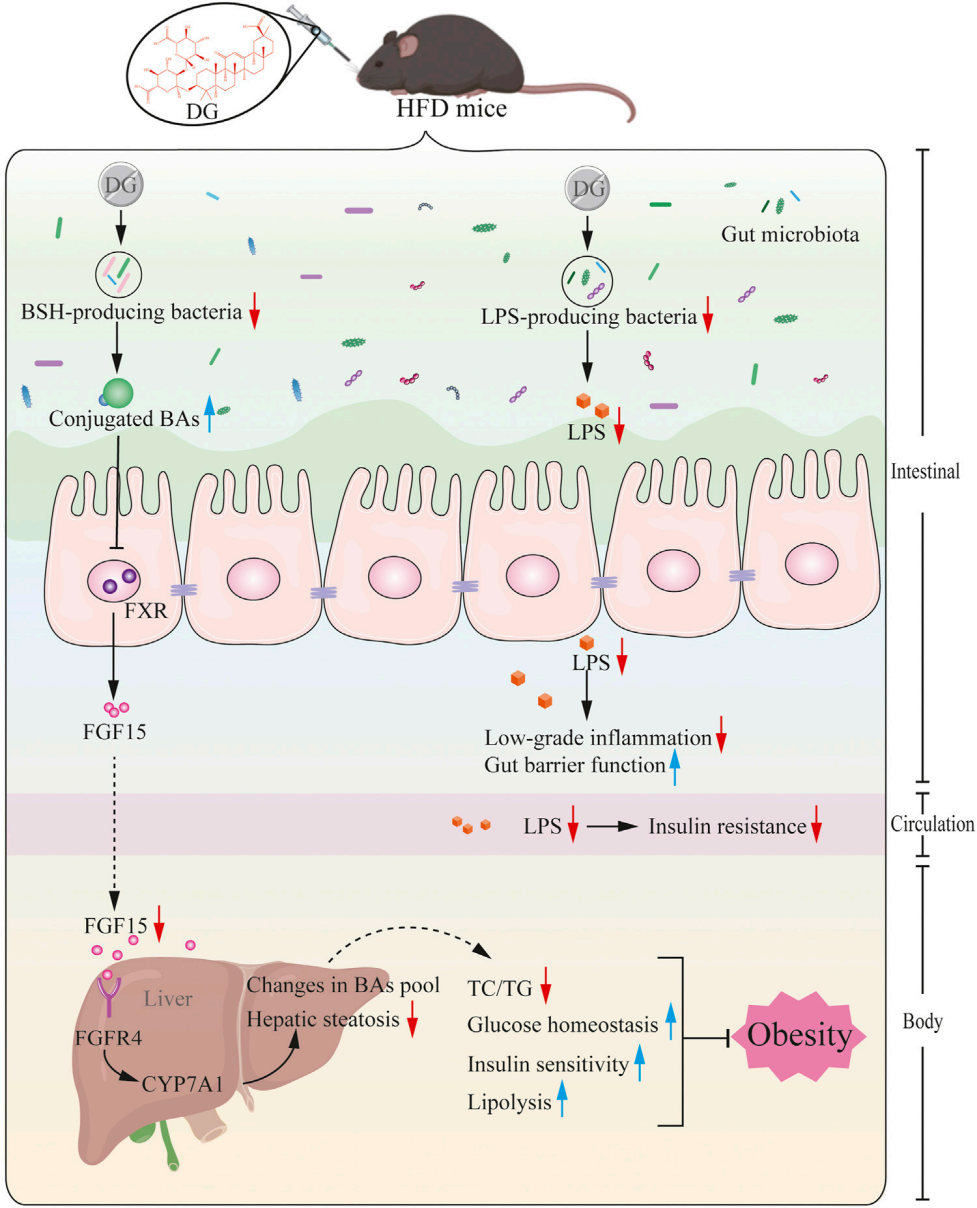
Potential mechanism for DG prevents against obesity through modulating gut microbiota in high-fat diet fed mice. Primary BAs, generated from cholesterol in liver, were secreted to intestinal tract by conjugating with taurine in mice, later, these conjugated BAs were hydrolyzed to produce unconjugated BAs by BSH enzymes generated from intestinal microbes. BSH enriched microbes was reduced by DG intervention causing increased levels of conjugated BAs, which suppressed ileal FXR-FGF15 feedback signaling, followed by repression of BA synthesis gene (CYP7A1) expression, leading to changes in BA profile, and ultimately brought a series of metabolic improvements including lower serum glucose and insulin levels, increased insulin sensitivity, few hepatic steatosis and resistance to weight gain. Moreover, DG treatment decreased LPS-producing genera resulting in decline of serum LPS level, inducing amelioration of colonic inflammation, intestinal barrier impairment and metabolic disorders like obesity and insulin resistance. Abbreviations: DG, diammonium glycyrrhizinate. BAs, bile acids. BSH, bile-salt hydrolase. FXR, farnesoid X-activated receptor. FGF15, fibroblast growth factor 15. CYP7A1, cholesterol 7α hydroxylase. LPS, lipopolysaccharide.

Based on the prior work discussed above and the evidence presented herein, we, therefore, rendered that a series of metabolic improvements produced by DG in obese mice induced by high fat diet were partially realized via modulating gut microbiota (Figure 9). In specific, it might thus work in two ways together. On one side, DG treatment increased the levels of conjugated BAs by decreasing the abundance of BSH-producing genera, and then repressed ileal FXR-FGF15 feedback signaling; on the other side, administration of DG reduced serum LPS levels and enhanced intestinal mucosal barrier by reducing LPS-producing genera.

In this present study, we explore the potential mechanisms behind DG intervention against obesity. And these preliminary studies partially explain how DG improves obesity-induced metabolic disorders via modulating gut microbiota. We conclude the efficacy of DG in the treatment of obesity mainly depends on inhibition of ileal FXR-FGF15 axis. This provides a potential molecular target for the clinical treatment of obesity and other metabolic disorders, further, it enables a possible pathway for the new drug development. Besides, a growing number of studies has paid more attention to the role of FXR in metabolic diseases due to the importance of its expression in metabolically active tissues such as the liver and small intestine. However, the function of FXR is more intricate. Experiments of FXR-deficient mice sometimes produce conflicting results. Fxr^−/-^ mice are prone to hyperglycemia and hypercholesterolemia when fed with normal diet (Ma et al., 2006). Moreover, these knock-out mice are more likely to develop intestinal inflammation and increase intestinal permeability (Fiorucci et al., 2021). In contrast, Fxr^−/-^ mice in Ldlr^−/-^ background fed with high-fat diet has a protective effect on diet-related obesity and atherosclerosis (Zhang et al., 2006). Some studies also proved that Fxr-deficient mice fed a high-fat diet or bred on a genetically obese background (ob/ob) seemed to prevent obesity and improve glucose homeostasis (Prawitt et al., 2011; Zhang et al., 2012; Parséus et al., 2017). In this case, our research appears to contradict the former and support the latter. And this paradoxical phenomenon may result from microbiome differences between the diet and the environment where animals are raised, which may lead to different phenotypes. Thus, FXR and its regulatory role in host health by gut microbiota deserves further research.

## Data Availability

The datasets presented in this study can be found in online repositories. The names of the repository/repositories and accession number(s) can be found below: https://www.ncbi.nlm.nih.gov/, PRJNA771931.

## Supplementary Material

Supplementary Figure S1 DG increased the proportion of BAs in FXR antagonists and mRNA expression of BA transporter in high fat diet mice. **(A)** Changes in differential BAs composition. **(B)** Relative mRNA expression of hepatic BSEP (*n*=5). **(C–E)** Relative mRNA expression levels of ASBT, OST*α* and OST*β* in ileum, respectively (*n*=5). Values were denoted as mean ± SEM. * *P* < 0.05; ** *P* < 0.01; *** *P* < 0.001. Abbreviations: DG, diammonium glycyrrhizinate. BAs, bile acids. FXR, farnesoid X-activated receptor. BSEP, bile salt export pump. ASBT, apical sodium dependent bile acid transporter. OST*α/β,* organic soluble steroid transporters *α/β.*
