# Supplementary figures and images for: Diammonium Glycyrrhizinate Ameliorates Obesity Through Modulation of Gut Microbiota-Conjugated BAs-FXR Signaling
Source: Front Pharmacol. 2021 Dec 21;12:796590. doi: 10.3389/fphar.2021.796590 (PMC8724542; doi:10.3389/fphar.2021.796590)

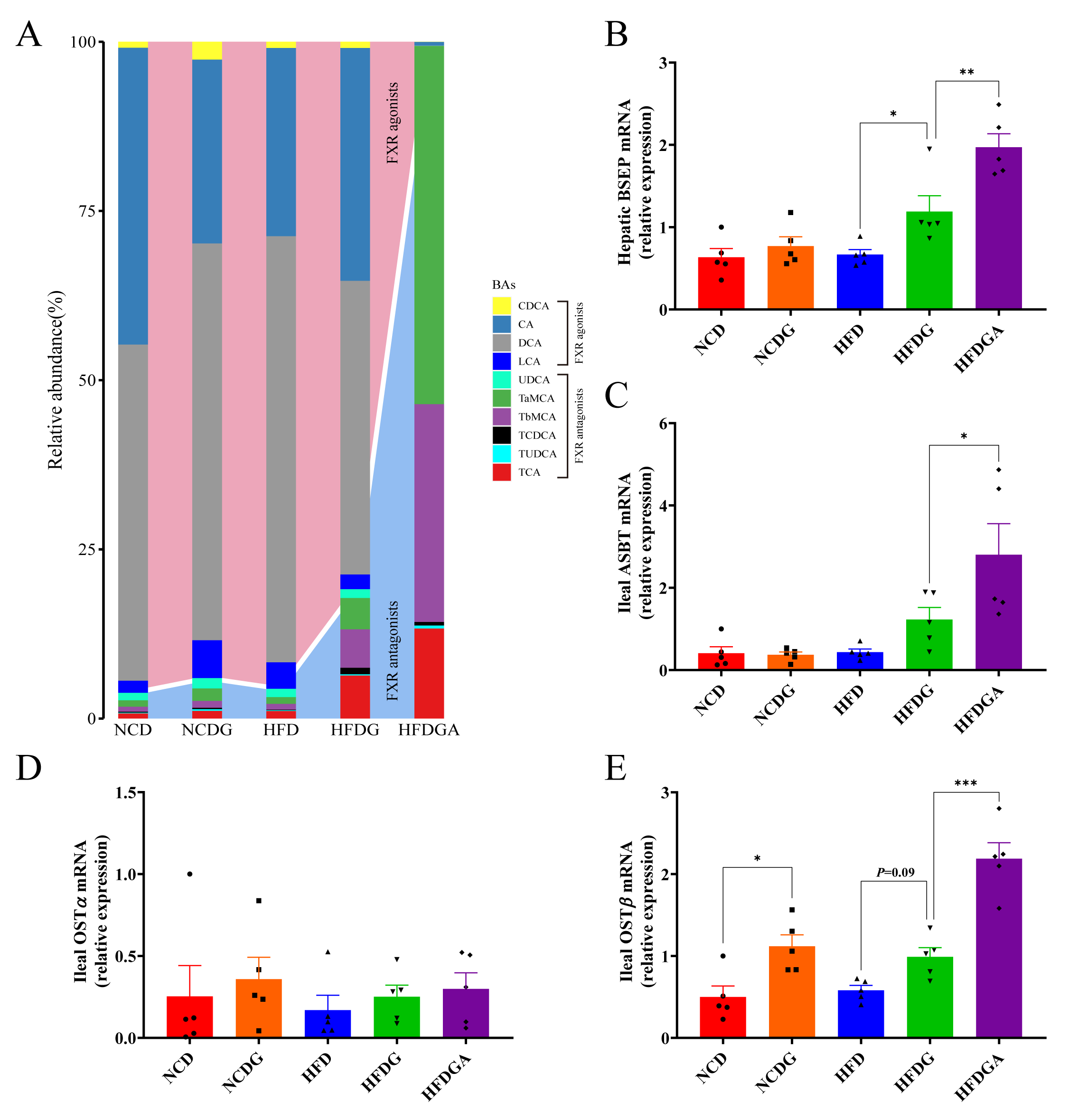

Supplement: Supplementary file 1 [file Image1.TIF]
